# The Probe Based Confocal Laser Endomicroscopy (pCLE) in Locally Advanced Gastric Cancer: A Powerful Technique for Real–Time Analysis of Vasculature

**DOI:** 10.3389/fonc.2019.00513

**Published:** 2019-06-13

**Authors:** Alessandra Capuano, Eva Andreuzzi, Eliana Pivetta, Roberto Doliana, Andrea Favero, Vincenzo Canzonieri, Stefania Maiero, Mara Fornasarig, Raffaella Magris, Renato Cannizzaro, Maurizio Mongiat, Paola Spessotto

**Affiliations:** ^1^Molecular Oncology, Centro di Riferimento Oncologico (CRO), IRCCS, Aviano, Italy; ^2^Pathology, Centro di Riferimento Oncologico (CRO), IRCCS, Aviano, Italy; ^3^Oncological Gastroenterology, Centro di Riferimento Oncologico (CRO), IRCCS, Aviano, Italy

**Keywords:** pCLE, gastric cancer, angiogenesis, angiogenic score, CD31, CD34, CD105, MMRN2

## Abstract

Probe based confocal laser endomicroscopy (pCLE) is an advanced technique which provides imaging of gastrointestinal mucosa at subcellular resolution and, importantly, a valid tool for the evaluation of microvasculature during endoscopic examination. In order to assess intratumoral vascularization and the efficiency of blood flow in locally advanced gastric cancer, we examined 57 patients through pCLE imaging. The vascular alterations in gastric cancer were mainly characterized by leakage and by the presence of tortuous and large size vessels. Defects in blood flow were detected very rarely. No association between the angiogenic score and the gastric tumor site or histological type was observed. Interestingly, no correlation was also found with the tumor grading indicating that the vascular angiogenic anomalies in gastric cancer represent an early pathological event to be observed and detected. The majority of patients displayed unchanged vascular alterations following neoadjuvant chemotherapy and this positively correlated with stable or progressive disease, suggesting that an unaltered angiogenic score could *per se* be indicative of poor therapeutic efficacy. Different vascular parameters were evaluated by immunofluorescence using bioptic samples and the vessel density did not correlate with clinical staging, site or histologic type. Interestingly, only CD105, Multimerin-2 and GLUT1 were able to discriminate normal from tumoral gastric mucosa. Taken together, these findings indicate that functional and structural angiogenic parameters characteristic of tumor blood network were fully detectable by pCLE. Moreover, the evaluation of tumor vasculature by real-time assessment may provide useful information to achieve tailored therapeutic interventions for gastric cancer patients.

## Introduction

Gastric cancer is a major leading cause of cancer-related deaths and a relative common malignancy (1). Surgical resection of the tumor represents the approved option to improve patients' survival (2). At diagnosis, most of the patients display locally advanced or metastatic disease. To improve their chances they are treated with palliative chemotherapy, including cisplatin, docetaxel, oxaliplatin and 5FU, among other drugs (3–5). Unfortunately, at 5 years from the diagnosis only 10% of the patients affected by advanced or metastatic gastric cancer will survive and the median overall survival (OS) is only 1 year (6). Therefore, new therapeutic approaches and more specific targeted therapies are required for the treatment of this type of cancer. Angiogenesis, the development of new blood vessels from pre-existing vasculature, affects tumor growth and the metastatic dissemination of cancer cells and in the latest years has gained attention in gastric cancer research as a promising target (7–9). The angiogenic process is regulated by a plethora of cytokines and growth factors as well as by different cell types (10). Tumor cells will not grow beyond the size of few millimeters unless induce the secretion of angiogenic factors and the development of blood vessel for nutrients and oxygen supply. However, the erratic angiogenic stimulus leads to the formation of an abnormal and not fully functional vascular network (11). An attractive approach that has been advanced in the latest years is the normalization of the aberrant tumor-associated vessels for the induction of a more efficient vasculature. A normalized vasculature would allow an improved delivery of drugs within the tumor and hence a better therapy efficacy (12, 13). One of the major regulators of angiogenesis is the VEGFA/VEGFR2 signaling axis and represents a major target for anti-angiogenic therapy (13, 14). Interestingly, in gastric cancer patients high VEGFA levels have been associated with reduced survival and increased tumor aggressiveness (15). Anti-angiogenic therapy in gastric cancer patients did not so far meet the expectations despite some improvements have been observed (16). Thus, in order to improve the efficacy of anti-angiogenic therapy, it is of major importance to better characterize the vasculature associated with this tumor type.

The probe based Confocal Laser Endomicroscopy system (pCLE) is a highly enhanced endoscopic technique constituted by a confocal scanning microscope integrated into a conventional flexible endoscope. pCLE provides high quality imaging of the tissue, with a resolution of approximately 1 micron of the mucosal layer (17, 18). The main clinical application for which pCLE was developed includes the real-time histopathological diagnosis of gastrointestinal lesions. However, in recent years additional potential application have been proposed and gained attention such as cancer screening on the basis of cellular and vascular changes (19–22). In fact, by using intravenously administered fluorescein sodium (23), images of vascular network can be clearly detected offering the possibility to gain information on the characteristics of gastrointestinal tumor vessels in real time (24). Our group was among the pioneers in exploiting this new promising imaging tool to analyze the angiogenesis pattern in patients with gastric and rectal cancer (25). Unlike conventional immunohistochemistry, the aim was to provide a prompt and accurate evaluation of the pattern and efficiency of intratumoral vessels through a non-invasive technique. The analyses suggested that pCLE was exceptionally powerful tool to visualize and characterize the tumor-associated vasculature. Interestingly, we found that rectal and gastric cancers were highly angiogenic; however, rectal tumors displayed a higher percentage of dilated vessels and presence of defective flow (25).

With the aim of developing new strategies to achieve more efficacious gastric cancer patient-tailored treatments, in this study we thoroughly analyze the vascular characteristics of this type of tumor evaluating the employment of pCLE in the assessment of intratumoral angiogenesis.

## Materials and Methods

### Patients

For this study 57 patients with locally advanced gastric cancer were enrolled to undergo endomicroscopy. Written informed consent was obtained from each patient on the day of the procedure. The methodologies conformed to the standards set by the Declaration of Helsinki. This study was approved by the Institutional Board of CRO-IRCCS, National Cancer Institute of Aviano (PN), Italy (IRB no. CRO-2014-03). The clinical evaluations are reported in Table 1. Patients underwent neoadjuvant multiregimen chemotherapy (oxaliplatin, capecitabine, and taxane) followed by surgical resection according to standard guidelines. Laboratory and pathological results were collected by means of the Hospital database.

**Table 1:** Clinic-pathological characteristics of patients.

	***N*^**°**^**	**%**
**GENERAL INFORMATION**		
All cases	57	100
Males	28	49
Mean age	62	
Females	29	51
Mean age	61	
**HISTOLOGIC TYPE (LAUREN CLASSIFICATION)**		
Diffuse	30	52.6
Intestinal	27	47.4
**TUMOR SITE**		
Body	35	61
Antrum	14	25
Fundus	5	9
Cardias	3	5
**STAGING**		
Ia-IIa	28	51
IIIa-IV	27	49

### Endoscopy Procedures and pCLE Analyses

pCLE analyses were carried out with GastroFlex UHD probe (Cellvizio, Mauna Kea Technology, Paris, France) during gastroscopy (Olympus series 180) and immediately before endoscopic ultrasonography (Olympus series 160) as previously described (25). Patients were examined before chemotherapy or surgical intervention (first pCLE). 13 patients (corresponding to 23% of all patients) were also examined after chemotherapy treatment (second pCLE). Images and sequences of the normal and neoplastic mucosa were taken and the conventional bioptic samples obtained with macrobiopsy (COOK Medical, Ireland) at the end of examination. Images were recorded within the first 10 min following i.v. injection of fluorescein (5 ml of a 10% solution). pCLE images were collected at 12 frames per second to assure high video quality and a direct visualization on a single erythrocyte scale. pCLE recordings were performed for at least 3 min resulting in a real-time imaging of more than 2 thousand frames. Using the videomosaicing function provided by the analysis software, we also obtained the reconstruction of the scanned panoramas of the mucosa. The mucosal architecture, vessel morphology and the efficiency of the blood flow were evaluated. The images were digitally stored and reviewed with the dedicated software package (Cellvizio Viewer, Mauna Kea Technologies) by a single investigator who was blinded to any clinical, endoscopic, or histopathological information. The angiogenic score was assigned on the basis of the presence of tortuous and large sized vessels, the vessels' leakage and the presence of defective flow as previously described (25).

### Immunofluorescence

For immunofluorescence (IF) analyses on bioptic samples, serial cryostat sections (7 μm) were collected on positively charged slides (BDH Superfrost Plus), air dried at room temperature (RT) and fixed with PFA for 15 min. After washing with phosphate-buffered saline (PBS), the slices were incubated with 0.5% Triton X-100 in PBS for 5 minutes at RT, saturated with 1% BSA−10% normal goat serum (DAKO) in PBS for 1 h at RT, and stained ON at 4 °C with the appropriate antibodies. Next, the samples were washed with PBS and incubated with the appropriate secondary antibodies and TO-PRO3 to stain the nuclei for 1 hour at RT. After washing with PBS, the slides were mounted in Mowiol containing 2.5% (w/v) of 1,4-diazabicyclo-(2,2,2)-octane (DABCO). Images were acquired with a Leica TCS SP8 Confocal system (Leica Microsystems Heidelberg, Mannheim, Germany), using the Leica Confocal Software (LCS). The monoclonal anti-human CD31, CD34, and CD105 antibodies were from Abcam (Cambridge, UK). The polyclonal anti-human Multimerin-2 (MMRN2) antibody was produced in our laboratories as previously described (26); the polyclonal anti-human GLUT 1 antibody was from Millipore (Temecula, CA, USA). The secondary antibodies conjugated with Alexa Fluor 488, 546 and TO-PRO-3 were from Invitrogen (Milan, Italy).

### Quantification Analyses

Fluorescence intensity and quantification were evaluated by means of the Volocity software Version 6.1.1 (PerkinElmer Inc.,Waltham, MA, USA). The expression of CD31, CD34, CD105, MMRN2, and GLUT1 was calculated as pixel positive area of at least four 40x acquired fields. Corresponding values were expressed as mean ± SD.

### Statistical Analysis

CD31, CD43, CD105, MMRN2, and GLUT1 expression levels between healthy and tumor mucosa of gastric cancer patients were compared by the Mann-Whitney non-parametric test. Relationships between positivity for vascular and angiogenesis markers and clinic-pathological features were evaluated using Spearman's rank correlation coefficient. Results were considered statistically significant for *P-*value < 0.05.

## Results

### pCLE Is a Valuable Tool to Assess the Properties of Gastric Cancer-Associated Vasculature

**Figure 1:**
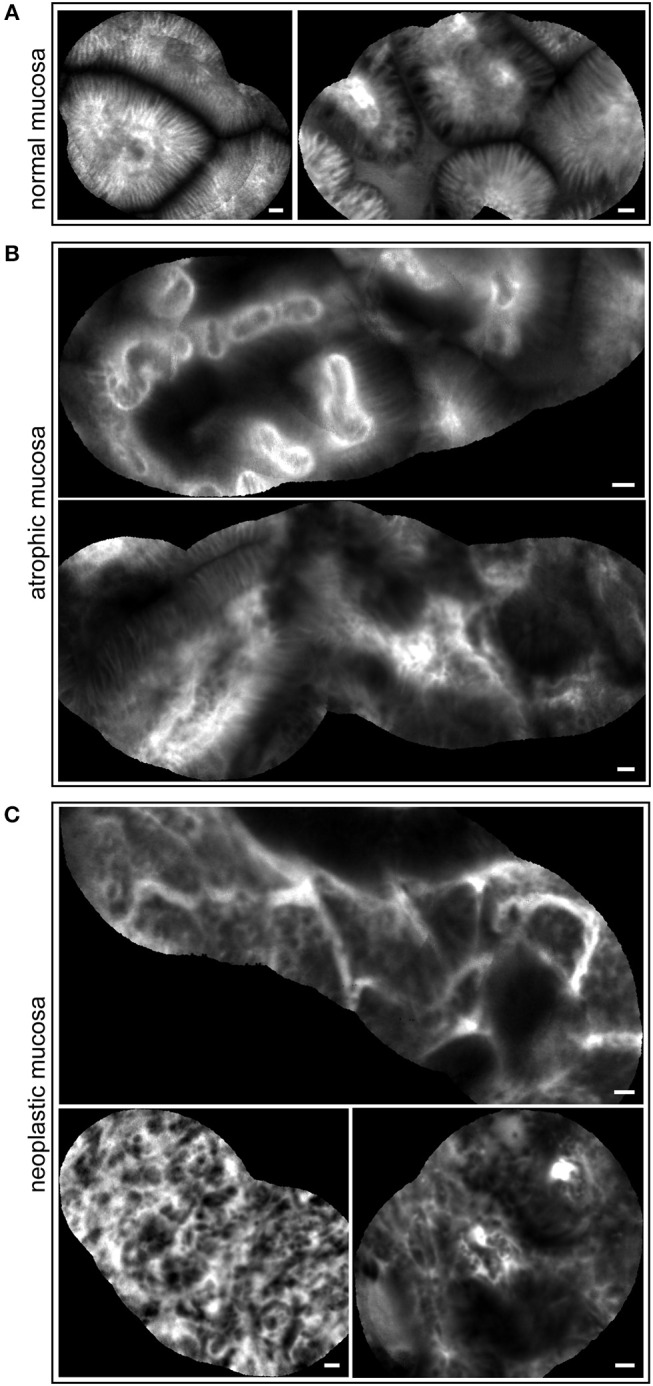
pCLE imaging in gastric mucosa. Representative mosaic reconstructions obtained from scanned panoramas of normal **(A)**, atrophic **(B)**, and tumor **(C)** gastric mucosa. The morphology of regular glands is well defined in **(A)**; some mild to moderate alterations in morphological epithelium architecture as well as unstructured vessels are easily observed in **(B)**; complexity of mucosa with severe cell irregularity and very altered vascular network are shown in **(C)**. Scale bar = 20 μm.

**Figure 2:**
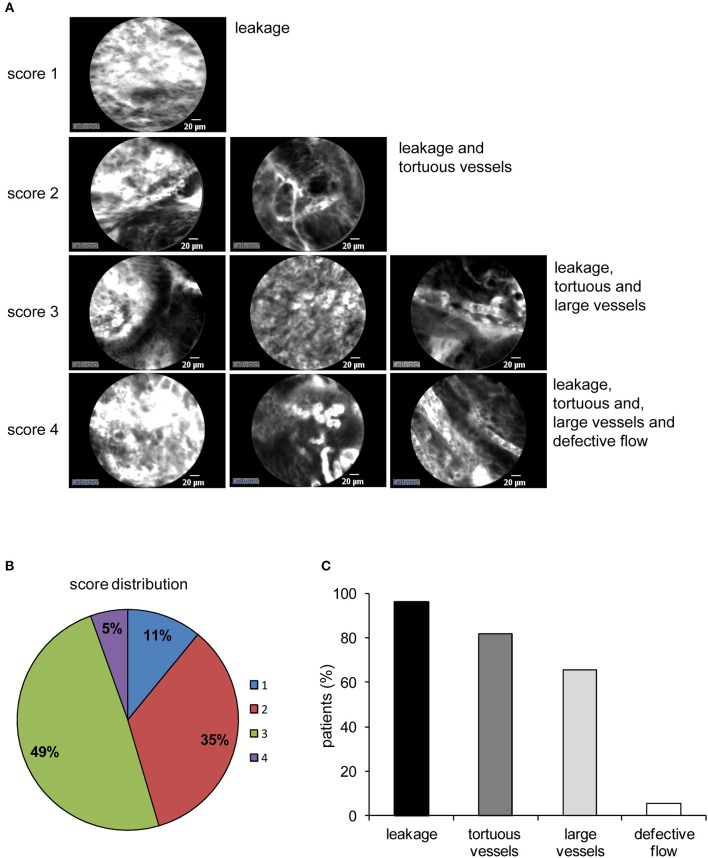
Angiogenic score in gastric cancer. **(A)** Representative images from patients displaying different angiogenic scores (from 1 to 4). The altered features of the tumor vasculature taken into account (leakage, tortuous and large vessels, and aberrant blood flow) are displayed (see also Videos S1, S2). A value of “1” was assigned to indicate the presence of each vessel feature and a value of “0” for the absence. The angiogenic score is the result of the arithmetical sum of the single features. **(B)** Distribution of the angiogenic score among all 57 gastric cancers analyzed. **(C)** Percentage of gastric cancer patients displaying the morphological and functional features as defined for the angiogenic score assignment. Aberrant blood flow was rarely detectable whereas leakage and the presence of tortuous vessels were the most frequent.

**Figure 3:**
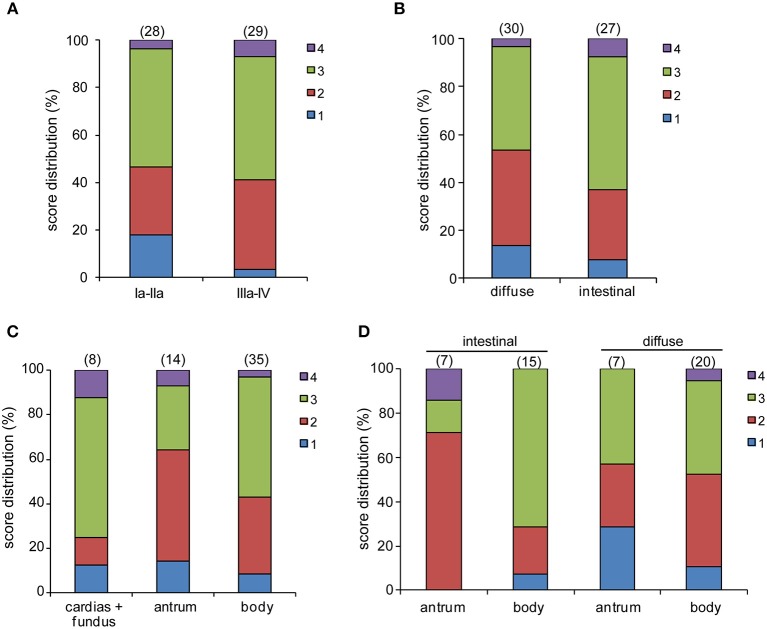
pCLE analysis and patient clinical categories. No statistically significant differences were observed between the score distribution and the tumor staging **(A)**, histologic type **(B)**, or tumor site **(C)**. Although not statistically significant, the percentage of patients with an intestinal type of gastric cancer and a score “3” is more associated with a body than an antrum localization **(D)**.

pCLE has been used in the diagnosis of gastric lesions for the possibility to easily detect with high accuracy the typical morphological alterations of the mucosal architecture (27–33). In Figure 1 we report representative reconstructions of the scanned panoramas of gastric mucosa obtained using the videomosaicing function which allows the alignment of the input frames. The differences in both morphological and vascular pattern between the normal (Figure 1A), atrophic (Figure 1B) and neoplastic (Figure 1C) gastric mucosa indicate that pCLE is a suitable tool not only for real time histopathological evaluations, but also for the assessment of the gastric cancer-associated vasculature. A thorough and prompt evaluation of the extent and quality of these vessels is in fact important to identify the patients that more likely will respond to anti-angiogenic treatment, as well as to develop new strategies to improve the efficacy of these treatments in non-responders. To this end we exploited the pCLE technology to evaluate the presence of tortuous and large-sized vessels, the presence of vascular leakage, and the efficiency of the vessels in terms of blood flow. We enrolled 57 patients affected by locally advanced gastric cancer and assigned an angiogenic score based on the pCLE analyses. The larger cohort of patients allowed us to confirm with statistical significance the observations gathered from few patients in a previous study aimed at comparing the vasculature of rectal and gastric tumors (25). Representative images of the vascular aberrations in gastric cancer patients characterized by different angiogenic scores are shown in Figure 2A (see also Videos S1, S2). A “3” angiogenic score was assigned to almost 50% of patients indicating that gastric tumors are characterized by a remarkably abnormal and unfunctional vasculature. In fact, a “1” angiogenic score was assigned to only the 11% of all the patients enrolled (Figure 2B). The most represented abnormalities were vessel leakage, which was detectable in 55 patients out of 57, the presence of tortuous vessels in 81% of the cases, and the presence of large diameter vessels in 67% of the patients. On the contrary, the presence of aberrant blood flow was detected only in 5% of the patients (Figure 2C and Video S2). To verify if the vascular pattern could depend on the clinical stage the patients were distributed into two categories: Ia-IIb and IIIa-IV. The statistical analyses indicated that there was no association between angiogenic score and staging (Figure 3A). In fact, the “2” and “3” angiogenic scores were homogenously distributed in both categories and the “1” and “4” angiogenic scores were not assigned to the low or high clinical stages, respectively. Next, we hypothesized that characteristics of the vasculature could depend on the tumor site or histological type according to Lauren classification (34, 35); however, we found that the angiogenic score did not correlate with these parameters (Figures 3B,C). A very high homogeneity was detected when we distributed the patients according to the histological type: no difference in angiogenic score distribution was observed between diffuse and intestinal type (Figure 3C). Although not statistically significant, the group characterized by lesions localized in the antrum displayed very frequently a “2” angiogenic score (Figure 3D). Taken together, the results from the pCLE analyses indicated that locally advanced gastric cancer is characterized by a remarkably abnormal and unfunctional vasculature.

### Neoadjuvant Chemotherapy Does not Significantly Affect the Vascular Properties

**Figure 4:**
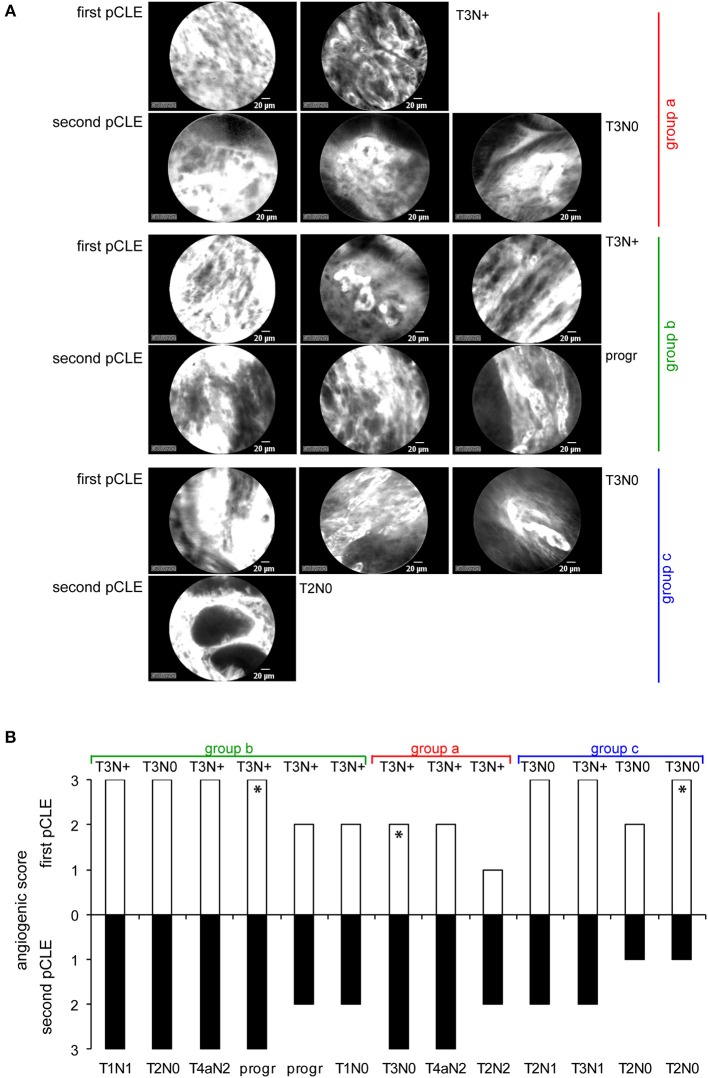
pCLE in patients after chemotherapy treatment. **(A)** Representative pCLE images collected from three patients belonging to three different groups displaying increased (group a), equal (group b) or decreased (group c) angiogenic score after therapy (second pCLE). **(B)** Graph showing the angiogenic score assigned before (first pCLE) and after therapy in 13 gastric cancer patients. Asterisks in the graph indicate the patients whose pCLE images were chosen as representative in **(A)**. For each patients the corresponding tumor grading or disease progression (prog) before and after therapy is reported both in **(A**,**B)**.

Among the enrolled patients a total of 13 (9 with T3N+ and 4 with T3N0, as evaluated during the first endoscopy) completed the neoadjuvant chemotherapy program. The subjects were re-evaluated by pCLE before surgery. These analyses indicated that the chemotherapy treatment did not significantly affect the angiogenic score of the tumors, despite a slight improvement in tumor grading was observed in almost 70% of these patients (Figure 4). These results suggest that neodjuvant therapy halted primarily the proliferation of tumor cells without affecting the properties and extent of the vascular network. Interestingly, these findings are in line with the fact that no correlation was found between the results gathered through the pCLE analyses and the tumor staging (Figure 3A). Given that locally advanced gastric cancers are characterized by a highly abnormal vasculature, it can be speculated that the slight efficacy of the treatment may depend on a poor delivery of chemotherapy within the tumor of stable or progressive disease patients.

### Gastric Cancer Vessels Are Poorly Efficient and Lead to Increased Intratumoral Hypoxia

**Figure 5:**
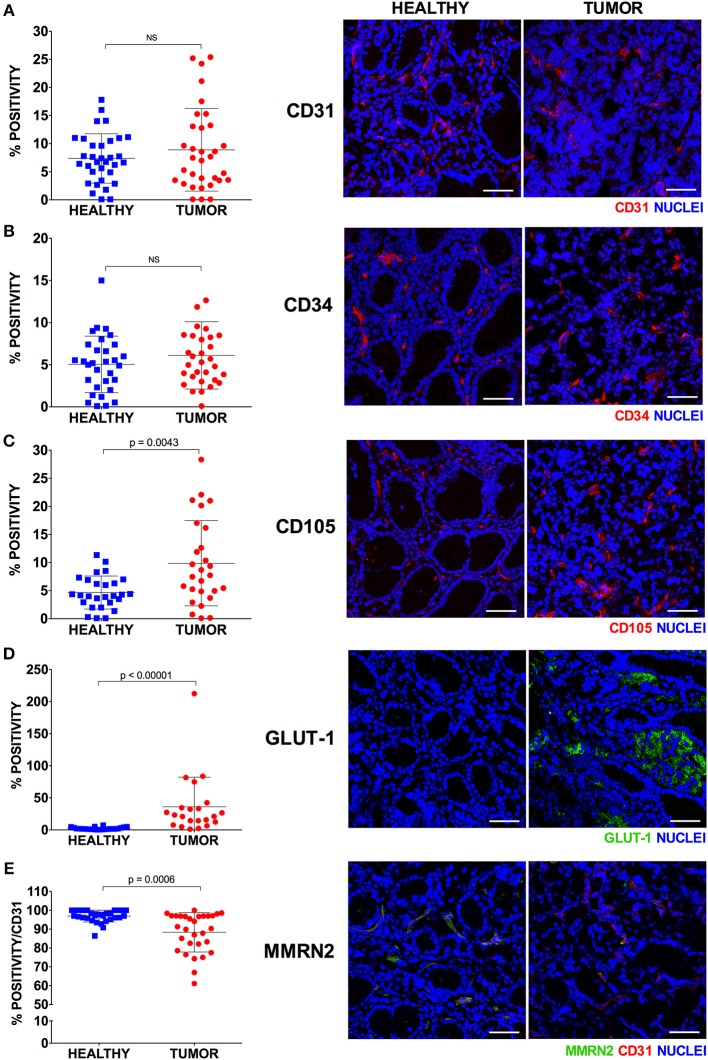
Expression of endothelial cell markers in healthy and tumor gastric tissues. Representative images of healthy and tumor gastric mucosa stained with anti-vascular marker antibodies and scatter plots of the corresponding expression calculated as percentage of IF positive stained area [CD31, **(A)**; CD34, **(B)**; CD105, **(C)**; GLUT-1, **(D)**; MMRN2, **(E)**]. For each marker at least four 40x magnified fields were evaluated. The results are expressed as mean ± SD. Scale bar = 50 μm.

**Figure 6:**
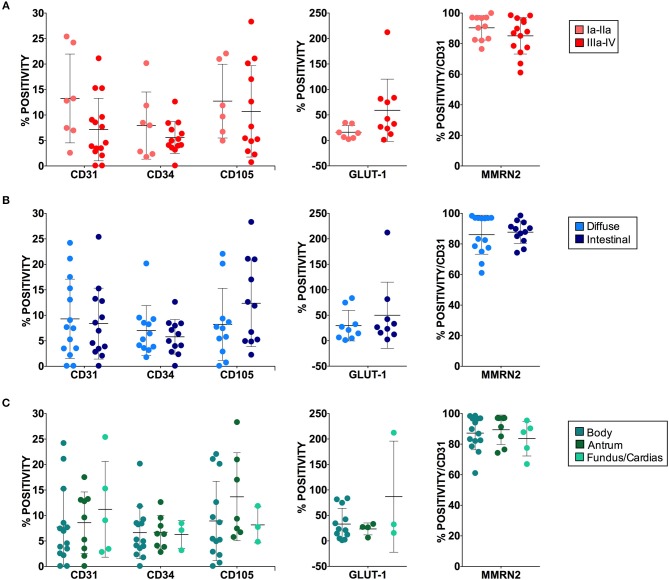
Angiogenesis markers and patient clinical categories. No statistically significant differences were observed between marker positive staining and tumor staging **(A)**, histologic type **(B)**, or tumor site **(C)**. For each marker at least four 40x magnified fields were evaluated. The results are expressed as mean ± SD.

In order to better define the quality of the gastric cancer associated vasculature we performed IF analyses employing different vascular markers i.e., CD31, CD34, CD105, Multimerin-2. Since Multimerin-2 was previously shown to affect HIF-1α expression (26) we also assessed the hypoxic levels in this tumor. To this end, we employed GLUT1 as a later maker of hypoxia, also in the light of the fact that it was recently reported to be a maker of poor prognosis (36, 37). To this end, during endoscopy and pCLE examination, bioptic samples of healthy and tumor mucosa of 33 patients were collected. First, we evaluated which marker could better detect the vascular density in normal and tumor gastric mucosa. These analyses are shown in Figure 5 where we report the percentage of positivity relative to the mucosal area for each vascular marker. The results from these analyses indicated that, despite the staining for CD31 and CD34 were higher in the tumor mucosa compared to the normal counterpart, the differences were not statistically significant. On the other hand, CD105 staining was significantly higher in tumor tissues, suggesting that this marker could better measure the vascular density in gastric tumors. We next assessed the expression of Multimerin-2, an extracellular matrix glycoprotein specifically expressed by endothelial cells exerting an angiostatic and homeostatic function (26, 38–41). We found a striking loss of Multimerin-2 expression in many gastric tumor associated vessels (Figure 5). Another parameter to verify, even if indirectly, the vascular efficiency is to measure the extent of hypoxic regions. To this end, the bioptic samples were stained with GLUT1. As reported in Figure 5, most of the gastric cancer samples displayed an increased expression of GLUT1 compared to the normal counterpart. The relative expression of these markers was independent from the tumor staging (Figure 6A), similarly to what observed with the angiogenic score determined by pCLE analyses (Figure 3A). Also, no correlation between the markers' expression and the histological type and tumor site was detected (Figures 6B,C).

These findings encourage the use of pCLE as a powerful tool to detect functional aberrations and other anomalies of the tumor vasculature rather than a mere microscopic system for the assessment of the vascular density.

## Discussion

In this study we assessed the possible employment of pCLE as a valid clinical tool to evaluate the vascular properties in gastric cancer. The analyses indicated that this endoscopic technique is indispensable to provide structural and functional insights useful to better characterize the tumor vascular network in locally advanced gastric cancer. Unlike the conventional immunohistochemical approach, which provides a static portrayal of the vasculature, pCLE is a technique able to dynamically visualize the efficiency of the vessels evaluating the extent of vascular leakage and also the efficiency of blood flow within the vessels. Despite markers of vascular leakage are available, such as the erythrocyte marker Ter119, the staining is difficult to interpret and often not reliable. In fact, the use of the Ter119 on the gastric cancer samples did not lead to any detectable specific staining (data not shown). On the contrary, the use of pCLE allows a clear detection of the fluorescein staining outside the vascular wall. It is thus possible to identify the percentage of vessels whose functionality is compromised and characterized by increased leakage. An additional information that can be provided exclusively with the use of pCLE is the efficiency of blood vessels flow. Despite not very frequently, the pCLE analyses in some occasions detected the presence of defective blood flow characterized by an erratic non-directional cell stream. It is conceivable that such vessels are unable to efficiently transport and distribute the blood as well the therapeutic drugs within the tumor, thus leading to increased hypoxia and decreased therapy efficacy, respectively. We subsequently compared the results from the pCLE analyses with those obtained with the use of different vascular markers following the immunohistochemical studies. We found that, unlike that of CD105, the analysis of the expression of CD31 and CD34 did not lead to a significant increased staining in gastric tumors, compared to the normal counterpart. Since CD105 is a marker of immature vessels, it is possible that these results depend on the fact that this maker is more appropriate for the detection of the newly formed vessels associated with tumors, as was previously suggested (42). In line with our observations it was also reported that CD34 was universally expressed in blood vessels within benign and malignant tissues, whereas CD105 expression was barely detectable in benign tissues and high in gastric carcinoma (43, 44). No clear role for CD34 in angiogenesis has been reported so far (45–48). On the contrary, it has been shown that CD105, as a receptor as TGF-β, may regulate the role of this cytokine in the angiogenic process and be more suitable for detecting newborn blood vessels in gastric and colorectal cancer (49). CD105 expression is strongly upregulated in various tumor tissues, including colon, breast, brain, lung, prostate, and cervix (50, 51). Little is known about the clinical significance of CD105 in gastric carcinomas; however, Nikiteas et al. have shown that VEGF and CD105 were involved in lymph node metastasis and acted as valuable indicators of the prognosis (52). The fact that high CD105 levels did not correlate with pCLE-based high angiogenic score may depend on the fact that vascular efficiency and stability is affected by several factors being controlled by cytokines, receptors and extracellular matrix components as well as mural cells. Thus, a comprehensive analysis of the proteins and pathways affecting vascular efficiency could be difficult and laborious to perform. On the contrary, the results obtained through the pCLE analyses provide information on the quality and efficiency of the vasculature independently from the molecular cause. In addition, an important advantage of the use of this tool is that the analyses precede the pathology investigations, thus providing a prompt information. We subsequently analyzed the expression of Multimerin-2, an extracellular matrix glycoprotein involved in the maintenance of vascular stability function. We found that many gastric cancer samples displayed loss of Multimerin-2. Since Multimerin-2 is a homeostatic molecule we hypothesize that vessels displaying low expression of this molecule are less efficient. However, no correlation was found between Multimerin-2 expression and the pCLE-based angiogenic score. This could be due to the fact that, as commented for the other vessel markers, vascular efficiency is affected by several molecules. In addition, the IF analyses are performed only in a small area of the tumor. Given the heterogeneity of the tumors it is likely that a more reliable analysis of the expression of the markers should be performed in several areas of the tumor. Interestingly, a stronger association was found between the results from the pCLE analyses and the expression of GLUT1. Indeed, the presence of abnormal, leaky and poorly perfused vessels, independently from the molecular cause, and the consequent lack of sufficient oxygen supply lead to increased hypoxia and the establishment of an exacerbated mutagenic tumor microenvironment (13). The vascular alterations recorded by pCLE as well as the expression of angiogenic markers did not correlate with clinical staging, histological type and tumor site. Our previous observations indicated that high angiogenic scores as assessed by pCLE analyses associated with tumor progression more in rectal than in gastric cancer patients (25). The larger cohort taken into account in this study confirmed and reinforced these observations.

To better characterize the vasculature associated with gastric cancer is a required clinical need not only to predict which patients will respond to anti-angiogenic therapy, but also to develop new strategies to overcome resistance. The anti-angiogenic drugs employed in gastric cancer clinical trials mainly target VEGFA such as the monoclonal humanized antibody bevacizumab, or the VEGFR2, in particular ramucirumab and selective tyrosine kinase inhibitors such as sunitinib, sorafenib and apatinib. According to a very recent systematic review and meta-analysis the addition of anti-VEGFR2 targeting agents to the first- or second-line chemotherapy could prolong patient's OS and PFS in advanced or metastatic gastric cancer (16). The study also highlighted the inefficacy of anti-VEGFA therapy in this type of tumor, unlike what observed in other type of cancers (53–56). Thus, to better characterize the quality of the gastric cancer-associated vasculature by pCLE may grant the possibility to predict which patients will be more likely to respond to anti-angiogenic therapy, sparing the others from costly ineffective treatments and side effects. In addition, the comparative results from the pCLE and IF analysis may open new avenues toward the development of new strategies to circumvent anti-angiogenic therapy resistance.

## Ethics Statement

This study was carried out in accordance with the recommendations of the Institutional Board of CRO-IRCCS, National Cancer Institute of Aviano (PN), Italy with written informed consent from all subjects. All subjects gave written informed consent in accordance with the Declaration of Helsinki. The protocol was approved by the Institutional Board of CRO- (IRB no. CRO-2014-03).

## Author Contributions

AC and EA developed the methodology, performed IF staining and graph editing, acquired data, and reviewed the manuscript. EP contributed to IF staining and analyses and critically reviewed the manuscript. RD collected, managed the bioptic samples, and reviewed the manuscript. AF collected data and helped for statistical analyses. VC provided final diagnosis and tumor staging. SM, MF, and RC identified and recruited patients and performed endoscopy and pCLE. RM helped in patient data managing. RC acquired funding, conceptualized the study and critically reviewed the manuscript. MM and PS conceptualized the study, reviewed as blinded investigators all pCLE videos, wrote original draft preparation and supervised investigation and analyses.

### Conflict of Interest Statement

The authors declare that the research was conducted in the absence of any commercial or financial relationships that could be construed as a potential conflict of interest.

## Supplementary Material

Video S1 Representative sequences of normal blood flow in a gastric cancer patient with an angiogenesis score of “2”.

Video S2 Representative sequences of abnormal flow in a gastric cancer patient with an angiogenesis score of “4”.
